# Therapeutic horizons in metabolic dysfunction–associated steatohepatitis

**DOI:** 10.1172/JCI186425

**Published:** 2025-07-01

**Authors:** Philip N. Newsome, Rohit Loomba

**Affiliations:** 1 Roger Williams Institute of Liver Studies, School of Immunology & Microbial Sciences, Faculty of Life Sciences and Medicine, King’s College London, Foundation for Liver Research and King’s College Hospital, London, United Kingdom.; 2 MASLD Research Center, Division of Gastroenterology and Hepatology, and; 3 Division of Epidemiology, Department of Family Medicine and Public Health, UCSD, La Jolla, California, USA.

## Abstract

Metabolic dysfunction–associated steatohepatitis (MASH), the progressive inflammatory form of MASLD, is now a leading cause of chronic liver disease worldwide. Driven by obesity and type 2 diabetes, MASH significantly increases the risk of cirrhosis, hepatocellular carcinoma, and liver failure. While public health interventions remain essential, therapeutic strategies targeting metabolic dysfunction, inflammation, and fibrosis are urgently needed. This Review focuses on pharmacological treatments in advanced development, including incretin-based therapies (GLP-1, dual, and triple agonists), metabolic modulators (PPAR, FGF21, and THR-β agonists), and novel agents such as fatty acid synthase inhibitors. Current regulatory approval is based on histological end points, with increasing interest in noninvasive biomarkers and personalized treatment approaches. Recent trials with agents such as semaglutide, tirzepatide, survodutide, lanifibranor, pegozafermin, and resmetirom demonstrate substantial promise in resolving MASH and improving fibrosis, but unresolved issues remain regarding treatment duration, response heterogeneity, and long-term adherence. Genetic variants (e.g., *PNPLA3* polymorphisms) and emerging molecular biomarkers may enhance stratification, while artificial intelligence is beginning to shape trial design and drug development. As the field moves toward combination therapies and precision medicine, the definition of therapeutic success will likely evolve to reflect both histological improvement and patient-reported outcomes. This Review provides a timely synthesis of the landscape, challenges, and future directions in MASH therapeutics.

## Introduction

Over the past 20 years, metabolic dysfunction–associated steatotic liver disease (MASLD) (1), formerly known as NAFLD, has become the commonest cause of liver disease in many parts of the world, largely due to the rise in the prevalence of metabolic dysfunction associated with obesity and type 2 diabetes mellitus. Current estimates of MASLD indicate a prevalence of 38%, which is expected to rise to 55% by 2040 (2). MASLD can progress to metabolic dysfunction–associated steatohepatitis (MASH), characterized by hepatic inflammation that predisposes the affected individual to fibrosis, cirrhosis, hepatocellular carcinoma (HCC), and liver failure. While the main response to this should be the adoption of comprehensive public health measures to prevent the development of obesity, these measures are difficult to enact and take many years to have an impact. Consequently, there have been concerted efforts to better understand the natural history of MASLD and MASH, the various phenotypes that exist within populations with these diagnoses, and the development of therapeutic approaches (3). Alongside the considerations of any given therapeutic compound (as a monotherapy or in combination), there are many other unresolved issues, including who to treat, when to treat, how long to treat, and, critically, how is success defined. This Review will principally focus on the relevant targets in MASH and the evolving classes of therapeutics that are being developed, with a discussion of current and future clinical end points and the shift toward personalized medicine.

## Clinical end points in MASH trials

Current regulatory end points in MASH trials are primarily focused on histological changes assessed via liver biopsy. These include resolution of MASH without worsening of fibrosis and improvement in fibrosis by ≥1 stage without worsening of MASH, both of which are accepted by the FDA and European Medicines Agency (EMA) as surrogate end points for accelerated approval. Additional end points in earlier phase trials include noninvasive biomarkers (e.g., alanine transaminase [ALT], aspartate transaminase [AST]), MRI–proton density fat fraction (MRI-PDFF) for liver fat quantification, liver stiffness via elastography, and composite histology scores, such as the NAFLD activity score. While these measures provide insight into disease activity and progression, limitations in reproducibility, accessibility, and correlation with clinical outcomes remain notable challenges.

## Weight loss pharmacotherapies

For most patients with MASH, due to coexisting overweight/obesity, there is a strong rationale for weight loss interventions to address not only their liver disease, but also the many related comorbidities. While there are many different weight loss pharmacotherapies such as orlistat (pancreatic lipase inhibitor), phentermine/topiramate, and naltrexone/bupropion, the most effective and widely used class are glucagon-like peptide-1 (GLP-1) agonists, used either alone or in combination with other incretin agents (4). These long-acting agonists stimulate insulin secretion from the pancreas in a glucose-dependent manner and also act on the hypothalamus to suppress appetite; therefore, they are widely used in the treatment of diabetes mellitus and obesity, with profound effects on weight loss and glycemic control. Alongside these benefits, GLP-1 agonists have been shown to be effective in cardiovascular outcome studies as well as in improving renal function.

For these reasons they have been studied in patients with MASH. Liraglutide (5), and then semaglutide (6), were studied in phase II clinical trials and were demonstrated to increase rates of histological resolution of NASH (now known as MASH) compared with placebo (39% and 59%, respectively, compared with 9% and 17% in the placebo arms). These effects were also seen alongside beneficial effects on weight and glycemic control. No improvements in liver fibrosis were seen, although there was reduced fibrosis progression alongside an improvement in noninvasive markers of liver fibrosis. Recently the effects of 2.4 mg semaglutide once weekly were studied in a phase III placebo-controlled randomized controlled registration trial in patients with MASH and stage F2/F3 fibrosis. Both regulatory approved primary end points of MASH resolution (62.9% vs 34.1%) and fibrosis improvement (37.0% vs 22.5%) were met, as were other statistically powered end points, such as the dual read-out of MASH resolution and fibrosis improvement (32.8 vs 16.2%) over 72 weeks of treatment (7). Semaglutide delivered improvements in cardiometabolic parameters and noninvasive markers of liver fibrosis in this study. Other GLP-1 agonists, such as exenatide and dulaglutide, have been shown to have positive effects on liver fat and liver enzymes (8, 9), but neither agent has been studied in a histological end-point trial. In a 48-week study using 2.4 mg semaglutide once weekly among patients with MASH cirrhosis, there were metabolic improvements but no improvement in either histological or imaging assessments of liver fibrosis (10).

Given the mechanism of action of GLP-1 agonists, their histological benefits are relatively predictable, although the beneficial effects on liver fibrosis in the recent phase III trial described above challenged the dogma about efficacy on this aspect of liver disease (7). The balance of evidence would suggest that, in the absence of hepatic GLP-1 receptors (11), any effect on fibrosis is likely to be largely indirect and mediated through a reduction in the upstream drivers of the condition. There remains speculation that GLP-1 agonists may exert a systemic antiinflammatory effect (12), independent of weight loss, that is mediated through action on circulating monocytes known to express GLP-1 receptors.

## Unresolved issues

### Duration of treatment.

Current studies have been up to 72 weeks in duration and have delivered high rates of MASH resolution (59%). Will longer treatment result in higher levels of MASH resolution such that all individuals ultimately respond or is there a ceiling effect from weight loss? Identifying which patients are likely to respond to GLP-1 therapy in terms of weight loss and also MASH will allow for a personalized medicine approach. Furthermore, once a liver benefit is observed, how much longer should treatment continue and at what dose level? Patients will likely regain weight once GLP-1 therapy is discontinued and hence reinstate the drivers of liver injury; therefore, strategies are required to address this, possibly by transitioning to a lower maintenance dose. Cessation of GLP-1 receptor agonist therapy is associated with substantial weight regain, with studies reporting patients regaining approximately two-thirds of lost weight within 1 year of stopping treatment (13). This highlights the chronic nature of obesity and the potential need for long-term or maintenance therapy to sustain benefits. Not only are the environment/lifestyle/economic circumstances underlying overweight/obesity often unchanged during the GLP-1 receptor agonist treatment period, but, in addition, weight loss upregulates appetite hormones. It is not clear if prolonged weight loss results in a resetting of appetite hormones or whether adjunctive therapies are required to achieve this.

### Adherence.

Studies of prescriptions and their renewals suggest relatively high levels of discontinuation of GLP-1 agonists after 6–12 months, which is presumed to relate to the range of gastrointestinal side effects that are associated with these therapies. Clarifying GLP-1 therapy discontinuation rates is important, as estimates vary depending on the data source. Real-world studies report discontinuation rates of 30%–50% within the first year (14, 15), often higher than those seen in clinical trials, where adherence is typically better due to close monitoring and support. It remains unclear what the longer-term adherence will be if these therapies are licensed for use in liver disease, where there may be a stronger motivation for patients to continue taking them. A combination of better education and support alongside the motivation of the clinical indication is likely to be critical, but data on longer-term adherence and maintenance of efficacy will be important. Oral GLP-1 receptor agonists are in ongoing development and may improve treatment adherence due to ease of administration, though current formulations have not yet achieved the same degree of weight loss as injectable agents.

### Nonresponse.

Genetic variants in the GLP-1 receptor and its signaling partner β-arrestin1 have been linked to reduced responsiveness to GLP-1 receptor agonists (16), which additionally highlights the potential role of pharmacogenomics in predicting treatment efficacy. An interesting observation with weight loss interventions is that while there is a clear link between weight loss and histological improvement at the aggregate level, there are often notable exceptions at the individual patient level. This likely reflects heterogeneity within the population diagnosed with MASH, and analysis of response to GLP-1 agonists across the many reported genotypes will be revealing. Understanding heterogeneity in response to GLP-1 agonists is relevant for patients with lean MASH, where the use of GLP-1 agonists may not be the most appropriate therapy.

### Use in patients with advanced fibrosis/cirrhosis.

Although studies thus far have not demonstrated an increased risk profile when GLP-1 agonists are used in a population of patients with advanced MASH, there are questions about these therapies’ effect on muscle loss and whether this muscle loss might precipitate liver decompensation. Recent studies have shown that reductions in lean muscle mass can account for approximately 20%–30% of total weight loss achieved with GLP-1 receptor agonists, raising concerns about potential sarcopenia, particularly in older or metabolically vulnerable populations (17). As sarcopenia is often seen in patients with advanced F3 fibrosis and is commonly seen in patients with cirrhosis, careful consideration is required in these settings as well. Close monitoring of body composition is therefore required with pharmacotherapy regimens with consideration given to simultaneous interventions that may preserve muscle mass potential such as resistance-based regimens.

## Dual and triple agonists

### Glucose-dependent insulinotropic polypeptide and GLP-1 receptor agonist.

Glucose-dependent insulinotropic polypeptide (GIP) agonism may have beneficial effects on the liver by improving insulin sensitivity, reducing inflammation, and modulating lipid metabolism. Preclinical studies also suggest that GIP may synergize with GLP-1 to enhance metabolic control and reduce hepatic steatosis (4). Tirzepatide, a once weekly dual agonist of GIP and GLP-1 receptors, is used for the treatment of both obesity and type 2 diabetes, leading to significant weight loss that is greater than seen with GLP-1 analogs alone (18). In a large multicenter phase IIb trial, 190 patients with biopsy-confirmed MASH and stage 2 or 3 fibrosis were randomized to 52 weeks of once weekly subcutaneous treatment with either tirzepatide (5 mg, 10 mg, or 15 mg) or placebo (18). In this study, tirzepatide led to significant improvement in the rates of MASH resolution, ranging from 62% to 46% in tirzepatide-treated patients versus 11% in placebo-treated patients, as well as significant improvements in ≥1 stage improvement in fibrosis, ranging from 51% to 55% in tirzepatide-treated patients versus 30% in placebo-treated patients, respectively. Tirzepatide demonstrated improvements in both imaging and serum biomarkers of liver fat, inflammation, and fibrosis (18–20). Further studies are needed to validate these findings in a larger and more diverse cohort of patients in a phase III trial before clinical use in patients with MASH.

### GLP-1 and glucagon-receptor agonists.

Glucagon receptor agonism may benefit MASH by increasing energy expenditure, promoting lipid oxidation, and reducing hepatic fat accumulation although precise mechanisms require further delineation. When combined with GLP-1 or GIP agonism, glucagon receptor agonists may enhance weight loss and metabolic improvements, contributing to greater reductions in liver steatosis and inflammation. There are several dual glucagon and GLP-1 agonists in clinical development for the treatment of MASH-related fibrosis, including cotadutide, pemvidutide, and survodutide. Given that hepatocytes to do not express GLP-1 receptors, the main action of GLP-1 in MASH likely relates to its effects in inducing weight loss, reducing appetite, and improving glycemic control. However, glucagon receptors are extremely well expressed in hepatocytes; therefore, combining glucagon-receptor agonists with GLP-1 analogs provides the additional direct, liver-centric effect of glucagon receptor agonism. This effect drives increased lipolysis and increased hepatic gluconeogenesis, leading to liver triglyceride mobilization and potentially increased energy expenditure. Together, dual agonists including cotadutide have shown significantly greater liver fat reductions than GLP-1 analogs alone, providing a theoretical rationale of higher potency on both MASH resolution and potentially fibrosis improvements (21). Survodutide, a once weekly subcutaneous dual glucagon–GLP-1 agonist, was examined in a large, multicenter, phase IIb trial in patients with biopsy-confirmed MASH and stage 2 or 3 fibrosis (22). 293 patients were randomized to either survodutide (2.4 mg or 4.8 mg or 6 mg) or placebo once weekly subcutaneously over 48 weeks. Survodutide was superior to placebo, with significant improvement in MASH activity ranging from 43% to 62% in survodutide-treated patients versus 14% in placebo-treated patients. There was also a higher number of patients with ≥1 stage improvement in fibrosis scores, ranging from 34% to 36% in survodutide-treated patients versus 22% in placebo-treated patients, respectively. Discontinuation rates were higher in patients randomized to the 6 mg survodutide arm, although dropouts often occurred early in the uptitration phase. Most common adverse effects seen were gastrointestinal in nature, with abdominal pain leading to drug discontinuation. A slower UP–titration protocol may be considered in future phase III trials to achieve a balance between efficacy and tolerability.

### GIP, glucagon-receptor, and GLP-1 receptor agonist.

Of particular relevance is retatrutide, a novel triple agonist that engages GLP-1, GIP, and glucagon receptors. In a recent phase II trial, retatrutide led to marked reductions in liver fat and improvements in metabolic parameters in patients with MASLD (23). These findings suggest that triple agonists may represent a promising therapeutic strategy for MASH by targeting multiple pathways involved in disease progression.

### Unresolved issues.

The scientific debate continues over whether GIP receptor engagement is best achieved through agonism or antagonism. Evidence indicates that tirzepatide exhibits a lower affinity for GIP receptors compared with native GIP, potentially functioning as a partial GIP antagonist (24). At higher doses, it may promote receptor downregulation over time. Amgen, on the other hand, supports a GIP antagonist strategy and is developing AMG-133, a molecule that combines GLP-1 agonism with GIP antagonism, for obesity treatment (NCT04478708). The interplay between receptor affinity and the balance of antagonism versus agonism likely influences biological outcomes, adding complexity to understanding GIP pharmacology. Long-term outcome studies will ultimately determine which approach is more effective and durable.

The optimal balance of glucagon and GIP agonism relative to GLP-1 agonism in combination therapies remains under active discussion. Cotadutide has an in vitro potency ratio of 5:1 for GLP-1/glucagon activity (25). Short-term clinical studies have shown it reduces liver fat by approximately 33% and offers glucose-lowering effects comparable to liraglutide at a dose of 1.8 mg. Survodutide (BI 456906) exhibits in vitro potency similar to native GLP-1, though it is about six times less potent than endogenous glucagon. This weekly agent has demonstrated clinically meaningful outcomes, including significant weight loss (up to 20% at the highest doses) and improved glucose control. In a Phase II randomized, double-blind, placebo-controlled trial, survodutide demonstrated significant efficacy in patients with MASH and fibrosis stages F1–F3. Over 48 weeks, patients receiving survodutide experienced improvements in MASH resolution without worsening fibrosis, with up to 62% achieving this primary end point compared with 14% in the placebo group. Additionally, up to 36% of survodutide-treated patients showed ≥1 stage improvement in fibrosis versus 22% with placebo. Liver fat content reductions of ≥30% were observed in up to 67% of the survodutide groups compared with 14% in the placebo group. The most common adverse events were gastrointestinal, including nausea, diarrhea, and vomiting, leading to discontinuation in 20% of survodutide-treated patients versus 3% with placebo. Consequently, survodutide is now being studied in a phase III trials for MASH (22). By contrast, pemvidutide has an in vitro activity ratio of 1:1 for GLP-1/glucagon, which may account for its superior hepatic fat reduction of up to 90% (26). However, this efficacy comes with a potential slight increase in blood glucose levels. Considering that type 2 diabetes is a common comorbidity in MASH, achieving potent liver fat reduction with dual agonists may require accepting a small but elevated risk of hyperglycemia at least early in the treatment, which may normalize with continued weight loss. Table 1 lists some of the key areas of research interest in GLP-1 use in MASH.

## Metabolic modulators

Metabolic modulators in the context of MASH refers to therapies that target key nuclear receptors and metabolic pathways involved in lipid metabolism, inflammation, and fibrosis. These include PPAR agonists (e.g., lanifibranor), FGF21 analogs (e.g., pegozafermin), and thyroid hormone receptor β (THR-β) agonists (e.g., resmetirom), which aim to correct underlying metabolic dysfunction independent of weight loss.

## PPARs

PPARs are nuclear receptor proteins that are important in the regulation of inflammation, glucose homeostasis, and lipid metabolism, all elements implicated in the pathogenesis of MASH (27). There are three main subtypes of PPARs, each with distinct roles in metabolic processes: PPARα is primarily expressed in the liver, heart, kidney, and muscles. It regulates the oxidation of fatty acids, promotes lipid breakdown, and decreases triglyceride levels by stimulating genes involved in fatty acid oxidation; PPARγ is predominantly found in adipose tissue. It is critical for adipogenesis and insulin sensitivity as well as regulating lipid storage and glucose metabolism alongside antiinflammatory properties. Finally, PPARδ is ubiquitously expressed, influences both lipid metabolism and inflammation, and also promotes fatty acid oxidation in muscle and liver as well as being involved in energy expenditure. Given these roles, several PPAR agonists, targeting different PPAR subtypes, have been studied in MASH. PPARα agonists such as the fibrates fenofibrate and gemfibrozil were primarily used to treat hyperlipidemia (28). They reduced hepatic triglycerides but had mixed results in reducing histological inflammation and fibrosis.

PPARγ agonists, often but not exclusively referred to as thiazolidinediones, include pioglitazone and rosiglitazone. By virtue of improving insulin sensitivity, these drugs are/were used to treat type 2 diabetes, but pioglitazone has also been studied extensively in MASH where it has shown consistent beneficial effects on liver steatosis, inflammation, and even fibrosis (29). Pioglitazone is also one of few drugs that has demonstrated histological improvement in patients with MASH, but despite meeting its primary end point (set at a lower bar than current regulatory standards) in the PIVENS trial, it did not impact MASH resolution (30). However, concerns about weight gain, fluid retention, and cardiovascular risks limited its use, and it has not been taken forward for more comprehensive evaluation. Emerging research on stereoisomers of pioglitazone suggests they may retain the liver-specific therapeutic benefits while minimizing PPARγ-related side effects such as weight gain and fluid retention (31, 32). Recognizing the potential of agonizing multiple PPARs, dual and pan PPAR compounds have been developed and tested in MASH. Elafibranor, a dual PPARα/γ agonist, showed some efficacy in a phase II histology study, especially in improving liver inflammation and steatosis, but it did not meet its primary end point in the pivotal phase III trial (RESOLVE-IT) (33). PXL065, is a deuterium-stabilized R-stereoisomer of pioglitazone, designed to retain the mitochondrial target effects associated with pioglitazone’s efficacy in MASH while minimizing PPARγ activation and its associated side effects. In a phase II trial, PXL065 demonstrated dose-dependent improvements in liver fat content and fibrosis markers, supporting its potential as a safer, liver-targeted therapy for MASH (31). Saroglitazar, another dual PPARα/γ agonist that is approved in India for the treatment of diabetic dyslipidemia and MASH, has shown potential in reducing liver fat content, inflammation, and fibrosis in early trials, and further research is ongoing (34). Of interest is the pan-PPAR agonist lanifibranor, which activates PPARα, PPARγ, and PPARδ. In its phase II trial, lanifibranor met its primary end point and resulted in MASH resolution (49% vs 22%) and improvement in liver fibrosis (48% vs 29%) for the higher dose of 1200 mg (35). It is now being evaluated in a phase III registration trial. Ultimately, pan-PPAR agonists, such as lanifibranor, may offer broader metabolic and antifibrotic benefits by simultaneously activating PPARα, -δ, and -γ pathways, but this multireceptor engagement may also increase the risk of class-related adverse effects such as weight gain and edema compared with more selective dual or single PPAR agonists.

## Unresolved issues

### Long-term efficacy and safety.

Many of the studies with PPAR agonists have been of relatively short duration, and long-term studies are therefore needed to assess whether the benefits of these drugs persist over time as well as evaluating their safety profile, particularly in terms of cardiovascular risk. Other aspects of the safety profile of PPAR agonists, particularly those with PPARγ activity, that warrant attention are anemia, likely from hemodilution, whereas previous concerns regarding bone loss seem not to be significant (36, 37). These adverse effects highlight the importance of careful patient selection and monitoring in the use of PPAR-based therapies for MASH.

### Monotherapy or combination therapies.

MASH is a multifactorial disease, and PPAR agonists may need to be combined with other agents, such as GLP-1 receptor agonists, fibroblast growth factor 21 (FGF21) analogs, or antifibrotic drugs, to achieve the best outcomes.

## FGF21

FGF21 is an endocrine hormone primarily secreted by the liver, with additional expression in adipose tissue and skeletal muscle, particularly in response to metabolic stress such as fasting, ketogenic diets, or mitochondrial dysfunction. It has a half-life of 1–2 hours and plays a key role in energy regulation as well as lipid and glucose metabolism. There are multiple FGF21 receptor isoforms (FGFR1c, FGFR2c, and FGFR3c), which are predominantly expressed in liver, adipose tissue, pancreas, and brain. The action of FGF21 in the liver is mediated through a family of receptors, including FGFR and a coreceptor protein called the β-klotho, which are collectively termed the FGFR–β-klotho protein complex. FGF21 binds directly to both proteins and this engagement leads to its downstream signal transduction.

Three FGF21 analogs are currently in phase IIb/3 clinical development for MASH-related fibrosis: efruxifermin, pegozafermin, and efimosfermin. All of these FGF21 analogs significantly reduce MRI-PDFF over 12–24 weeks of treatment compared with placebo and have been shown to increase the key adipocytokine serum adiponectin (38). Furthermore, they improve hepatic insulin sensitivity, lower plasma triglycerides and LDL cholesterol, and inhibit DNL. The most common adverse effects seen with this class of drugs were gastrointestinal.

Efruxifermin, a long-acting Fc-fusion FGF21 analog, activates FGFR1c, FGFR2c, and FGFR3c in both liver and adipose tissue. In the Harmony trial (Phase IIb study), 128 patients with MASH stage 2 or 3 fibrosis were randomized to either weekly sub-cutaneous efruxifermin (50 mg or 28 mg) or placebo for 24 weeks (38). 50 mg Efruxifermin was better than placebo in both improving ≥ 1 stage of fibrosis without worsening of fibrosis (efruxifermin-treated groups had a response ranging between 39%–41% versus 20% in the placebo group) as well as resolution of MASH without worsening of fibrosis (efruxifermin-treated groups had a response ranging between 47%–76% versus 15% in the placebo group) (38), respectively. These participants then continued treatment for a total of 96 weeks, demonstrating sustained improvements in both fibrosis regression as well as MASH resolution.

Pegozafermin, a long-acting glycopegylated form of FGF21, binds to both FGFRs and β-klotho in liver and adipose tissue. In the phase IIb Enliven trial, 222 patients were randomized to different doses of pegozafermin or placebo administered subcutaneously weekly (or every 2 weeks) for 24 weeks (39). Pegozafermin was better than placebo in improving ≥1 stage of fibrosis without worsening of fibrosis (pegozafermin-treated groups had a response ranging between 22% and 27% versus 7% in the placebo group) as well as resolution of MASH without worsening of fibrosis (pegozafermin-treated groups had a response ranging between 23% and 37% versus 2% in the placebo group), respectively. Both efruxifermin and pegozafermin are currently in phase III trials, and we expect top line results in the next 1–2 years. Efimosfermin, a once-monthly fusion protein based on human IgG and FGF21, activates FGFR1c, FGFR2c, and FGFR3c in both liver and adipose tissue. In a phase IIb study, 84 patients with MASH stage 2 or 3 were randomized to receive either monthly 300 mg efimosfermin or placebo subcutaneously for 24 weeks. Efimosfermin was better than placebo in both improving ≥1 stage of fibrosis without worsening of MASH (efimosfermin-treated group had a 45% response versus 21% in placebo group) and resolution of MASH without worsening of fibrosis (efimosfermin-treated group had a 68% response versus 29% in placebo group), respectively (40).

## THR-β agonists

There are several THR-β agonists in clinical development for the treatment of MASH-related fibrosis and cirrhosis. These act by improving mitochondrial efficiency, thereby enhancing β-oxidation, reducing hepatic fat and hence lipotoxic stress, which attenuates stellate cell activation, potentially leading to improvements in MASH activity and fibrosis. Steroid hormone binding globulin (SHBG) levels typically increase in a dose-dependent manner and provide evidence of target engagement within one-week of dosing with THR-β agonists. Furthermore, MRI-PDFF responses provide a dose-dependent response of clinical efficacy over 12–24 weeks of treatment with this class of agents.

Resmetirom, a liver-selective oral THR-β agonist, has recently been conditionally approved by the FDA for the treatment of significant and advanced fibrosis due to MASH without cirrhosis, using a weight-based oral daily dose of either 80 mg or 100 mg. In a large, multicenter, randomized-placebo controlled trial, 966 patients with biopsy-confirmed MASH and either stage 2 or 3 fibrosis were randomized (1:1:1) to either 100 mg or 80 resmetirom or placebo over 52 weeks of treatment (41). MASH resolution without worsening fibrosis was significantly higher in the 100 mg group (30%) and 80 mg group (26%) compared with the placebo group (10%). Fibrosis improvement by ≥1 stage without worsening of NAFLD activity score was also significantly higher in the 100 mg group (26%) and 80 mg group (24%) compared with the placebo group (14%). The main adverse effects related to resmetirom were diarrhea and nausea, but overall it was well-tolerated and the severe adverse events were not different between the three groups. Resmetirom, as with other agents in this class of drugs, also reduced low-density cholesterols as well as lipoprotein a levels. These data suggest that THR-β agonists may not only improve the risk of progression of liver disease but also reduce the risk of cardiovascular disease in patients with MASH. These would have important implications in improving the overall burden of cardiometabolic-renal-liver complications in patients with MASH. Among predictors of response to resmetirom, the MRI-PDFF response criteria rule developed by Loomba and colleagues remains a useful tool, as those who achieve ≥30% relative decline in MRI-PDFF over 12–24 weeks have a more than five-times higher odds of developing a histologic response (42). It is important note that the phase III program for resmetirom is still ongoing, with extended follow-up and histological and clinical end points, alongside an open-label cohort of patients with compensated cirrhosis. In addition to resmetirom, other THR-β agonists such as VK2809 and ASC41 are currently under evaluation for MASH, with early-phase trials showing promising effects on liver fat reduction and metabolic parameters.

## Fatty acid synthase inhibition

De novo lipogenesis (DNL) is an important mechanistic pathway associated with the pathogenesis of MASH, and inhibition of DNL via inhibition of fatty acid synthase (FAS) has been linked to improvements in MASH. Emerging data from phase IIa and, more recently, phase IIb trials have demonstrated that treatment with denifanstat, an oral FAS inhibitor, improves MRI-PDFF and serum ALT levels in patients with MASH-related fibrosis. In a multicenter, phase IIb trial, 168 patients with MASH stage 2 or 3 were randomized (2:1) to either 50 mg denifanstat or placebo treated over 52 weeks. Denifanstat was better than placebo in both MASH resolution as well as fibrosis improvement (43). The most common adverse event was hair thinning, but overall, denifanstat was well tolerated and there was no imbalance in severe adverse events. Larger phase III trials are being planned.

## General perspectives on combination therapies

Given the complexity of MASH, which involves multiple pathways, including obesity, lipid metabolism and adipose dysfunction, inflammation, insulin resistance, and fibrosis, targeting just one pathway may be insufficient to halt or reverse the disease progression. Indeed, there may be synergistic effects from combining drugs with different mechanisms of action leading to greater efficacy than monotherapy.

## Current approaches to combination therapies in MASH

There are many combination approaches currently in clinical development, and, due to space constraints, we will focus on agents that are either being evaluated in phase IIb trials or for which there are already phase IIb data available. Table 2 provides how we think about mechanisms of action, their potential effect of liver histologic improvements, and their association with the likelihood of histologic response and choice of primary outcome depending on whether one expects MASH resolution or fibrosis improvement or both.

### Metabolic modulators and antiinflammatory agents.

Pioglitazone and vitamin E have been combined in small trials and have shown benefit in improving some histological outcomes; however, these findings have not been validated in larger trials with robust histological end points (30, 44, 45).

### Combination of acetyl co-A carboxylase inhibitor plus farnesoid-x receptor agonist with or without apoptosis signal-regulating kinase 1 inhibitor.

The ATLAS trial included patients with bridging fibrosis and cirrhosis due to MASH and examined the efficacy of monotherapy versus various combination approaches, including firsocostat (an acetyl co-A carboxylase [ACC] inhibitor), cilofexor (an farnesoid-x receptor [FXR] agonist), or selonsertib (an apoptosis signal-regulating kinase 1 [ASK-1] inhibitor) over 48 weeks. It found that a combination of ACC plus cilofexor demonstrated numerically higher improvements in improvement in MASH activity but did not demonstrate significant improvements in fibrosis (46). This was one of the earliest large, randomized controlled trials to examine the different mechanistic pathways and their potential to improve histologic end points in MASH. Currently there are no plans to pursue these drugs as part of a combination therapy in MASH trials. Given that obeticholic acid, an FXR agonist, did not receive FDA approval due to concerns of potential hepatoxicity and increased risk of gall bladder events, there is dampened enthusiasm to develop an FXR agonist in the treatment of MASH. Selonsertib monotherapy failed to improve fibrosis in phase III program, so that program has also been abandoned.

### GLP-1 receptor agonists and FGF21 analogs.

A large, international randomized placebo-controlled trial of FGF21 plus semaglutide versus either alone is underway to examine the clinical utility of a combination in patients with MASH stage 2–4 fibrosis in improving fibrosis stage by ≥1 stage. These data will help inform whether the combination of complementary mechanisms by way of FGF21 and a GLP-1 analogs will augment fibrosis regression particularly in patients with stage 3 and 4 fibrosis.

### Semaglutide, firsocostat, and cilofexor.

A large phase IIb trial is underway to examine the efficacy of semaglutide with an ACC inhibitor and an FXR agonist. A previous phase IIa trial demonstrated that there were greater improvements in AST and markers of fibrosis with the triple combination than with either therapy alone, providing a mechanistic rationale to pursue this approach.

When considering combination therapies in MASH, there are several considerations, as indicated below.

### Regulatory and developmental barriers.

The regulatory approval of combination therapies is complex, as it usually requires demonstrating the safety and efficacy of each component as well as the combination. Additionally, pharmaceutical companies may be reluctant to develop combination therapies if the components are produced by different manufacturers, leading to potential intellectual property and marketing challenges.

### Drug interactions and safety.

Combining multiple drugs increases the risk of adverse effects and drug-drug interactions and, therefore, careful evaluation must be given to the safety profile of each drug, particularly in patients who may already be on multiple medications for comorbid conditions (e.g., diabetes, cardiovascular disease). The safety of long-term combination therapy also needs to be thoroughly evaluated.

### Cost and accessibility.

Combination therapies can be expensive, and thus the cost-effectiveness of these treatments must be considered, especially given the high prevalence of MASH and the long duration of treatment required to see meaningful results. Accessibility to combination therapies may also be limited by insurance coverage and healthcare infrastructure.

### Clinical trial design.

Designing clinical trials to evaluate combination therapies in MASH is complex. Trials must be large enough to detect meaningful differences between treatment groups, and they will need to include diverse populations to ensure that the findings are generalizable.

## Personalized medicine

**Figure 1:**
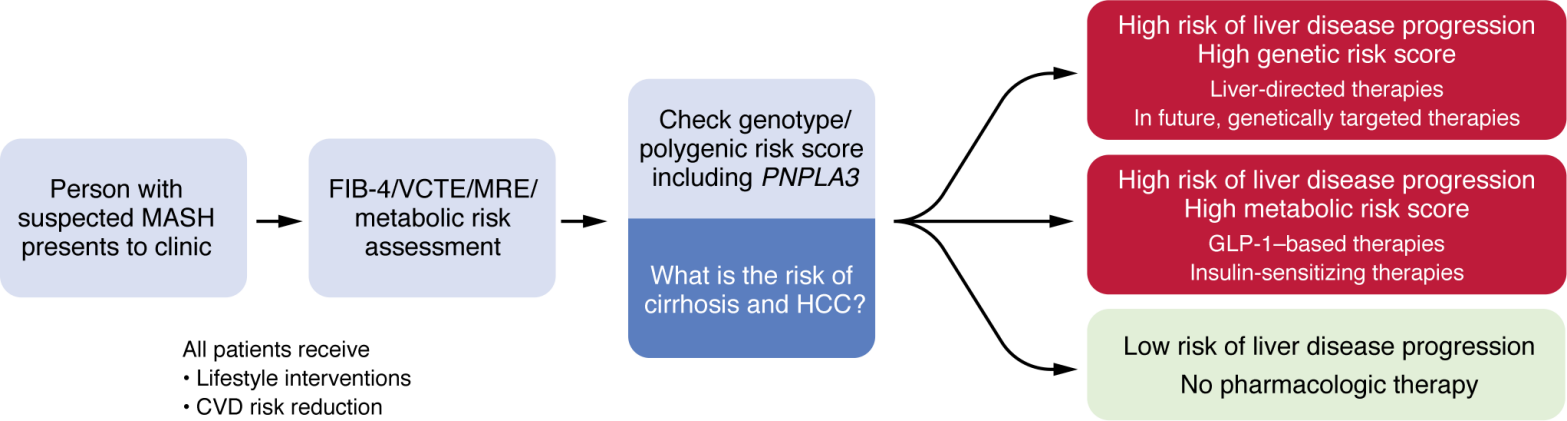
Precision medicine paradigm for MASH treatment from a genetic viewpoint. This figure illustrates a proposed personalized medicine approach for patients with MASH, starting with simple and inexpensive assessments of fibrosis and metabolic risk before moving to genomic stratification. FIB-4, fibrosis 4 index; VCTE, vibration-controlled transient elastography; MRE, magnetic resonance elastography; CVD, cardiovascular disease.

Recent work has proposed the existence of two distinct endotypes of MASLD, cardiometabolic and liver specific, based on clinical, genetic, and molecular profiling, with potential implications for personalized treatment strategies (47). Incorporating such phenotypic stratification may complement polygenic risk score approaches and enhance precision medicine efforts in MASLD. Indeed, patatin-like phospholipase domain-containing protein 3 (*PNPLA3*) genetic polymorphisms are associated with increased risk of liver disease progression, cirrhosis, and HCC (48–50), and emerging data suggest that *PNPLA3* may play a role in differential response to GLP-1 based regimens in MASH. Polygenic risk scores may also be potentially utilized to stratify patients into those who may have increased genetic risk versus those who may have increased metabolic risk (51). In the future, genetic risk score, including *PNPLA3*, and other single nucleotide polymorphisms may be utilized clinically to determine which therapies may be considered first-line treatment depending upon the genotype of the patients (Figure 1). Further studies are needed in this domain to better understand the clinical utility of polygenic risk scores in the assessment of steatotic liver disease. The integration of nongenetic biomarkers, such as transcriptomic, proteomic, and metabolomic signatures, offers a promising avenue for personalizing treatment in MASH by enabling more precise patient stratification and therapy selection (52). Additionally, artificial intelligence is being applied across MASH drug development to improve histological assessment (e.g., AIM-NASH), optimize patient recruitment, and predict treatment responses, ultimately enhancing trial efficiency and therapeutic precision (53).

## Future perspectives

The approval of new therapies is dependent on them meeting success criteria as outlined by the FDA/EMA. This consists of provisional approval based on a histological outcome, with full approval based on meeting clinical end points. Histological end points are challenging in terms of cost, patient acceptability, and variability in their interpretation due to sampling issues and pathologists’ evaluation. On the other hand, clinical end points are limiting due to the long duration of studies, and, therefore, the associated cost and time delay as well as the required commitment by patients. Ultimately, the field requires the validation of noninvasive biomarkers, which allow for shorter studies without recourse to liver biopsy. Success in trials could therefore be defined by a combination of biomarker improvements and imaging changes that correlate with histological improvements; as such, a decrease in liver stiffness, measured by elastography alongside a reduction in serum fibrosis markers, could serve as a noninvasive surrogate for histological fibrosis improvement. Moreover, it may be that the primary goal of noninvasive tests in trials shifts from predicting or indicating the degree of fibrosis to directly predicting clinical outcomes, as shown in the recent Lin et al. study on Agile 3+ (54). Incorporation of patient-reported outcomes, such as fatigue, general health, pain, and psychological well-being, could become a critical component of defining success in MASH trials, but these outcomes are unlikely to be sufficiently specific and quantifiable to be included as critical decision tools. Nonetheless, demonstrating an improvement in quality of life may be more meaningful to patients than changes in liver histology alone.

**Figure 2:**
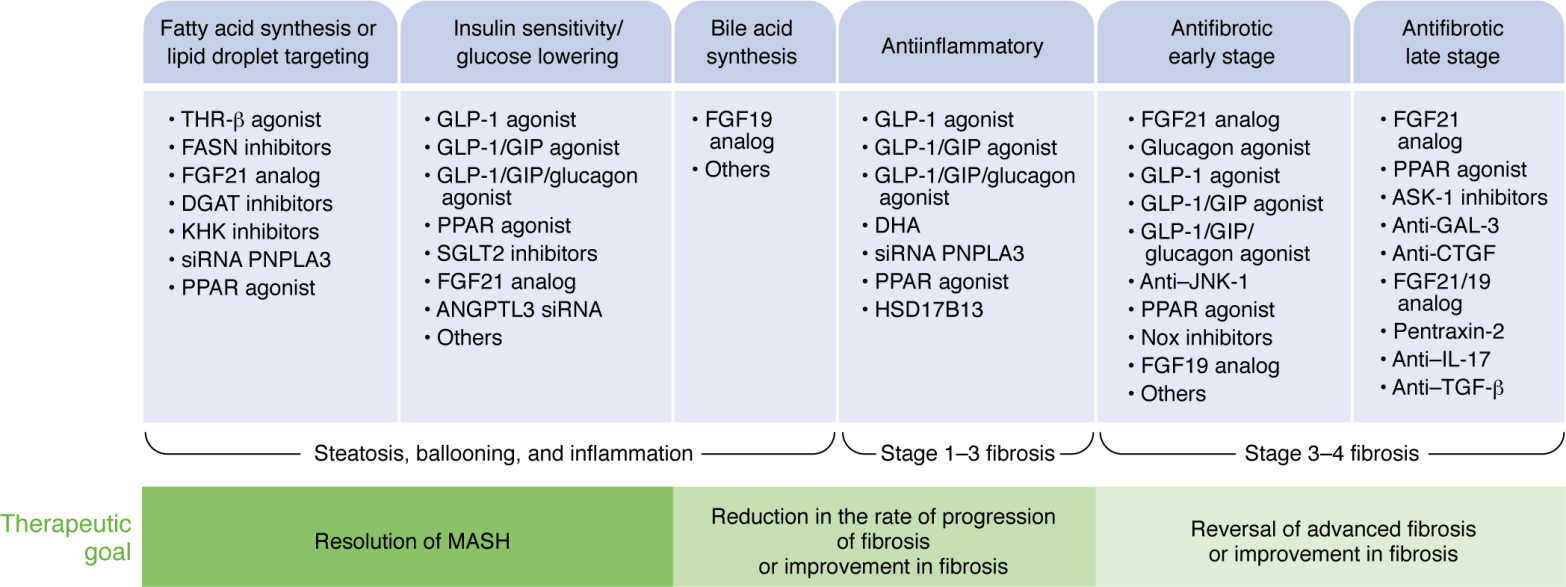
MASH therapeutic targets categorized by mechanism of action and subsequent biological and clinical efficacy in MASH. This figure highlights the broad range of therapeutic modalities under consideration for MASH and their categorization by broad mechanism of action. The mechanism of action, in turn, is a major determinant of which aspects of MASH will be affected and, therefore, informs how such therapies may be utilized. DGAT, diacylglycerol O-acyltransferase; KHK, ketohexokinase; DHA, docosahexaenoic acid; ASK-1, apoptosis signal regulating kinase 1; GAL-3, galectin 3.

Another perspective is that given MASH is a heterogeneous disease, with patients differing in terms of the severity of liver involvement, metabolic comorbidities, and risk of progression we should consider utilizing personalized read-outs of success. For example, in patients with early-stage disease, success might be defined as preventing progression, whereas in those with advanced fibrosis, success could be defined as regression of fibrosis or preventing cirrhosis. Moreover, the creation of metabolic dysfunction–associated steatotic liver disease as a new field to encompass both metabolic dysfunction and alcohol-related liver disease will also impact therapeutic approaches; many of the drugs discussed herein are of relevance to that area of study. Furthermore, a personalized medicine approach might tailor therapy based on genetic, epigenetic, or biomarker profiles that can stratify patients based on their likelihood of response to specific therapies, and trials could define success differently for each subgroup (Figure 2).

## Conclusion

The future of defining success in MASH trials, and therefore approval of new therapies, will hopefully involve a shift from single, histology-based end points to more comprehensive, multidimensional approaches that better address the complexity of the disease and its impact on patients. This will include a greater emphasis on noninvasive biomarkers, clinical outcomes, patient-reported outcomes, and the durability of treatment effects. Ultimately, the goal is to define success in a way that is meaningful to patients, reflects long-term benefits, and aligns with personalized treatment strategies.

## Figures and Tables

**Table 2:**
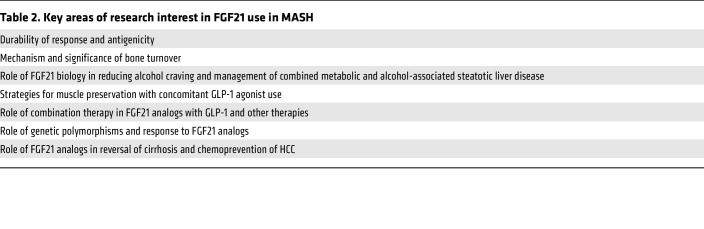
Key areas of research interest in FGF21 use in MASH

**Table 1:**
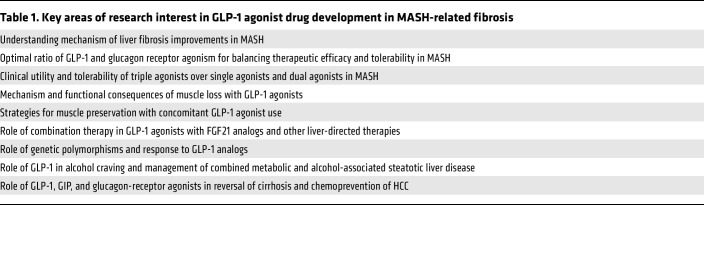
Key areas of research interest in GLP-1 agonist drug development in MASH-related fibrosis

## Version 1 07/01/2025

Electronic publication
